# Spatial and temporal adaptation of predictive saccades based on motion inference

**DOI:** 10.1038/s41598-020-62211-8

**Published:** 2020-03-24

**Authors:** Takeshi D. Itoh, Ryuji Takeya, Masaki Tanaka

**Affiliations:** 1 Department of Physiology, Hokkaido University School of Medicine, Sapporo, 060-8638 Japan; 2 Present Address: Graduate School of Science and Technology, Nara Institute of Science and Technology, Ikoma, 630-0192 Japan

**Keywords:** Decision, Saccades, Decision

## Abstract

Moving objects are often occluded behind larger, stationary objects, but we can easily predict when and where they reappear. Here, we show that the prediction of object reappearance is subject to adaptive learning. When monkeys generated predictive saccades to the location of target reappearance, systematic changes in the location or timing of target reappearance independently altered the endpoint or latency of the saccades. Furthermore, spatial adaptation of predictive saccades did not alter visually triggered reactive saccades, whereas adaptation of reactive saccades altered the metrics of predictive saccades. Our results suggest that the extrapolation of motion trajectory may be subject to spatial and temporal recalibration mechanisms located upstream from the site of reactive saccade adaptation. Repetitive exposure of visual error for saccades induces qualitatively different adaptation, which might be attributable to different regions in the cerebellum that regulate learning of trajectory prediction and saccades.

## Introduction

In daily life, small moving objects (such as birds or cars) are often temporally occluded behind larger stationary objects (such as trees or buildings) in front of them, but we can easily predict the location and timing of their reappearance. Extrapolation of object trajectory and prediction of timing require the integration of multiple processes for visual motion, spatial working memory and perception of elapsed time. Along with the object kinematics, several cognitive factors can alter the spatiotemporal estimate of object reappearance from behind an occluder^1–5^.

Consistent with these behavioral findings, an inference of object motion activates many brain regions including and beyond the visual cortices, such as the superior temporal cortex, the prefrontal cortex, the posterior parietal cortex and the lateral cerebellum^6–8^. In experimental animals, visual motion sensitive neurons in these cortical and subcortical regions continue firing during target occlusion in the absence of self-motion^9–13^. Given that the cerebellum is involved in both motor and non-motor cognitive functions through reciprocal connections with the association areas in the cerebral cortex^14–18^, the cerebro-cerebellar network might be suited to the maintenance of the internal model of object motions.

In the motor systems, the cerebellum is critically involved in adaptive learning^19,20^. The cerebellum helps compute prediction errors of goal-directed movements and updates internal forward models for predictive motor control^21,22^. Previous studies also suggested a role for the cerebellum in adaptive control of non-motor cognitive functions^23^. Using the inferred motion paradigm, a recent study demonstrated that subjects with cerebellar degeneration were able to accurately predict the timing of the target’s reappearance from occlusion but were unable to adapt temporal prediction when the target reappearance was systematically shifted in time^24^. Another study provides a realistic computational model of the cerebellum that can account for many empirical features of temporal learning^25^. Based on these previous observations, we hypothesized that the cerebellum might represent internal models of object trajectory that can be recalibrated through learning.

For a better understanding of the role of the cerebro-cerebellar network in sensory prediction, we trained monkeys to generate predictive saccades to target reappearance from behind an occluder and then attempted to induce adaptation of trajectory prediction. Our data show that spatial and temporal adaptation can occur and takes place upstream of adaptation of visually triggered reactive saccades.

## Results

### Spatial and temporal adaptation of predictive saccades based on motion trajectories

**Figure 1:**
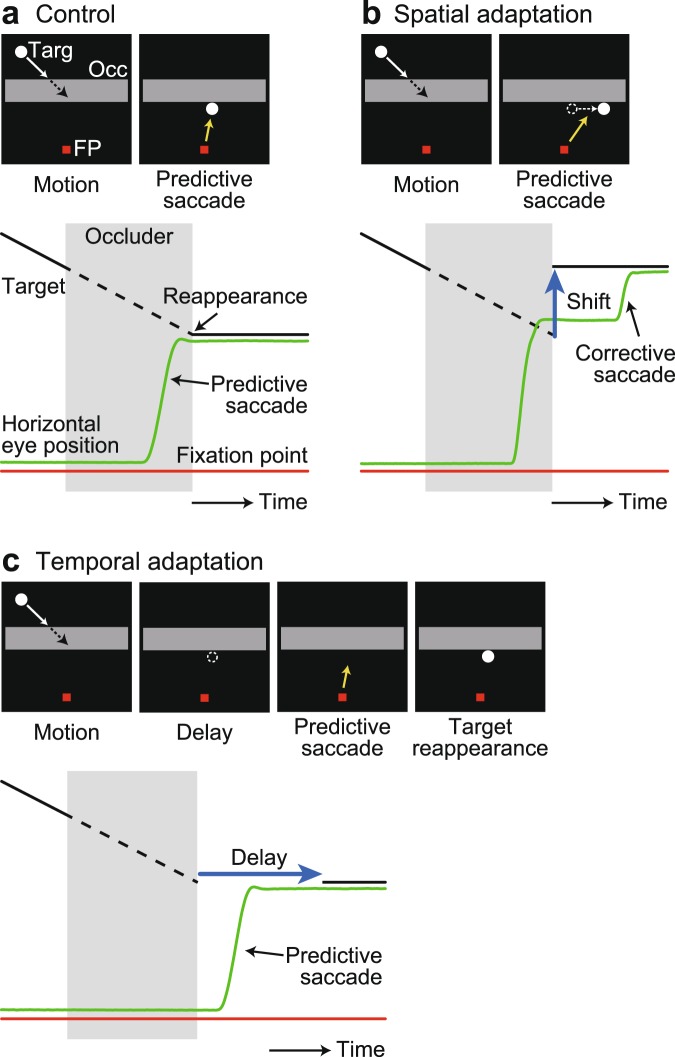
Predictive saccade task. (**a**) Control trial. While monkeys looked at the fixation point (FP), a white target spot (Targ) appeared on the upper half of the screen and moved at 20°/s. After 500 ms, the target was occluded behind a stationary occluder (Occ, 10° height) that was visible throughout the trial. The target reappeared at the bottom side of the occluder and remained stationary. Monkeys were required to make a predictive saccade to the location of target reappearance within ±150 ms to obtain a liquid reward. Note that the FP remained visible at the time of saccade initiation. (**b**) In the spatial adaptation paradigm, the location of target reappearance was shifted 5° horizontally. (**c**) In the temporal adaptation paradigm, target reappearance delayed or advanced by 200 ms.

Three macaque monkeys were trained in the predictive saccade task (Fig. 1a, Methods). Each trial began with the appearance of a red fixation point (FP, 0.5° square) presented below a gray rectangle (10° height, ‘occluder’). During eye fixation, a white target spot (0.5° circle) appeared above the occluder and moved obliquely at 20°/s. After a 500-ms excursion, the target reached the upper side of the rectangle and was occluded for 580–710 ms. For each trial, the target path was randomly selected among 28 combinations of initial target location and trajectory angle (see Methods). Animals were trained to make a predictive saccade to the position where the target would reappear on the lower side of the occluder. To induce adaptation, the location or timing of target reappearance was altered in a block of trials (Fig. 1b,c).

**Figure 2:**
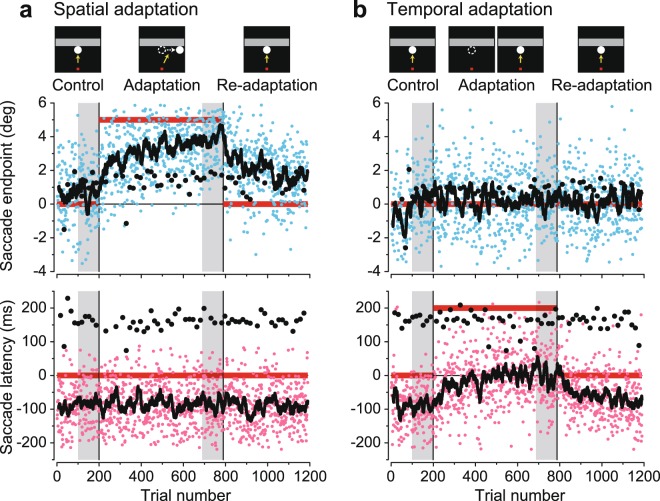
Time course of adaptation. (**a**) Spatial adaptation experiment. Upper panel plots the relative location of saccade endpoint as a function of trial number. Zero on the ordinate indicates the horizontal location of target reappearance predicted from the target trajectory before occlusion. Trials were presented in three different blocks comprising control, adaptation and re-adaptation blocks. Blue dots indicate individual predictive saccades for target reappearance. Black dots indicate visually guided saccades directed toward the target that suddenly appeared on the bottom side of the occluder in a small fraction of trials. The black trace indicates the moving average of 15 consecutive predictive saccades. Horizontal red lines indicate the locations of target reappearance. The lower panel plots saccade latencies relative to the expected timing of target reappearance. Pink dots indicate the data for individual prediction trials, while black dots indicate those for visually guided saccades. Horizontal red lines denote the timing of target reappearance. Note that the endpoints of visually guided saccades (upper panel) and the latencies of predictive saccades (lower panel) remained unchanged during adaptation of predictive saccades. (**b**) Temporal adaptation experiment. Conventions of the graph are the same as in a. Note that the predictive saccade latencies gradually changed (lower panel) while their endpoints remained unchanged (upper panel). Predictive saccades generated >80 ms following target reappearance were excluded from further quantitative analysis (0.13% and 0.25% trials in the experiments shown in a and b, respectively; see Methods).

Figure 2a shows the time courses of horizontal saccade endpoint and latency in a representative spatial adaptation experiment in monkey W. In this experiment, the location of target reappearance (upper panel, horizontal red bar) was shifted 5° rightward during the adaptation block (delimited by two vertical lines). The data clearly show that individual saccade endpoints relative to the expected location of target reappearance (blue dots) and their running averages (15 consecutive trials, black trace) gradually moved toward the actual location of target reappearance (horizontal red bar). To quantify the level of adaptation, we compared saccade endpoints during the last 100 trials between the first and the second blocks (gray shaded areas). In this particular experiment, saccade endpoints changed by 3.27° (unpaired *t*-test, *t*_188_ = −13.6, *p* < 10^−3^), which corresponded to an adaptation gain of 0.65 (for a 5° shift). Conversely, when we compared saccade latencies before and during adaptation (lower panel, pink dots and black line), no significant change was found (mean difference: −3.5 ms, *t*_188_ = 0.36, *p* = 0.72). Additionally, both the endpoints and latencies of visually guided saccades in the last 10 probe trials in the block remained unchanged (black dots, mean differences: 0.55° and 4.8 ms, unpaired *t*-test, *t*_18_ = −1.7 and −0.38, *p* = 0.12 and 0.71 for the endpoints and latencies, respectively). When we computed the degree of adaptation transfer from the predictive to the visually guided (reactive) saccades by comparing the changes in saccade endpoints, the value was 0.17 for this experiment. The transfer of saccade adaptation was also evaluated by computing regression coefficient for the changes in predictive and reactive saccade endpoints during adaptation (Methods); the value was 0.060 for the experiment.

The animals could also adapt to changes in the timing of target reappearance. Figure 2b shows the data from another representative temporal adaptation experiment performed in the same monkey (W). When the timing of target reappearance was delayed by 200 ms during the adaptation block, saccade latencies were gradually altered (lower panel, pink dots), whereas saccade endpoints remained unchanged (mean difference: −0.27°, upper panel, unpaired *t*-test, *t*_186_ = 1.1, *p* = 0.30). On average, saccade latencies during the analyzed intervals differed by 93 ms between the first and the second blocks (unpaired *t*-test, *t*_186_ = −8.5, *p* < 10^−3^), which corresponded to adaptation gain of 0.47 (for 200 ms delay). Again, both the endpoints and latencies of visually guided saccades remained unchanged (black dots, mean differences: 0.48° and −14 ms, unpaired *t*-test, *t*_18_ = −1.2 and 1.2, *p* = 0.26 and 0.24, respectively).

**Figure 3:**
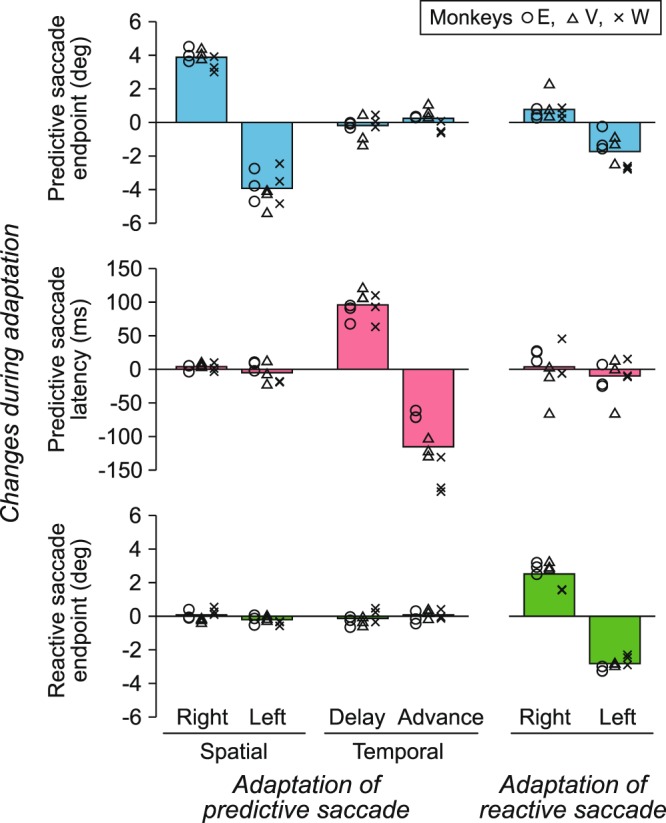
Quantitative summary. Effects of adaptation were quantified by computing the changes in saccade endpoints or latencies measured during the last 100 trials in the first and the second blocks (shaded areas in Figs. 2 and 4). The left two columns indicate the data for predictive saccade adaptation and the right column indicates the data for visually guided saccade adaptation. Different symbols indicate different monkeys. Each animal performed three experimental sessions for each condition (18 experiments for each monkey). Each bar indicates the mean of nine sessions (three sessions × three animals). Note that adaptation of predictive saccades occurred separately for the spatial and temporal changes in target reappearance (left two columns). Note also that adaptation of reactive saccades moderately altered endpoint of predictive saccades (upper right panel).

For each animal, we conducted three adaptation experiments for each direction of spatial (rightward or leftward) and temporal (delay or advance) adaptation (12 sessions in total). Figure 3 summarizes the data from three monkeys. During the spatial adaptation experiments, the endpoints of predictive saccades were shifted by 3.88° ± 0.51° (SD, *n* = 9, paired *t*-test, *t*_8_ = 22.6, *p* < 10^−3^) and −3.93° ± 0.94° (*t*_8_ = −12.6, *p* < 10^−3^) for the rightward and leftward adaptation experiments, respectively (Fig. 3, top left panel). The latency of predictive saccades remained unchanged during adaptation in both directions (mean differences: 4.0 and −5.0 ms, paired *t*-test, *t*_8_ = 1.8 and −1.1, *p* = 0.10 and 0.32, respectively, Fig. 3, left panel in the second row). Rightward adaptation of predictive saccades did not alter either the endpoint or latency of visually guided saccades (mean differences: 0.09° and 6.9 ms, *t*_8_ = 0.97 and 1.7, *p* = 0.36 and 0.14, respectively, Fig. 3, left panel in the third row), whereas leftward adaptation slightly but significantly altered reactive saccade endpoint (−0.20° ± 0.25°, *t*_8_ = −2.4, *p* = 0.043) and latency (12 ± 11 ms, *t*_8_ = 3.2, *p* = 0.013). On average, the amounts of gain transfer from predictive to reactive saccades were 0.023 ± 0.070 (SD, *n* = 9) and 0.051 ± 0.064 for rightward and leftward adaptation, respectively. We obtained similar results when adaptation transfer was measured as regression slopes for changes in saccade endpoints during the course of adaptation (0.0093 ± 0.037 and 0.036 ± 0.041 for rightward and leftward adaptation, respectively).

During these experiments, both the angle and the location of target trajectory were randomized in individual trials (28 combinations, see Methods). To examine the possible confounding effects of the target trajectory on the degree of spatial adaptation of predictive saccades, we performed a two-way ANOVA (Table 1). For the rightward adaptation experiments, the amount of adaptation tended to be greater when the angle of target trajectory was shallower (that is, with a longer occlusion interval) and when the location of target reappearance was closer to the screen center. This trend was absent for the leftward adaptation experiments.

**Table 1:** Effects of target trajectory on the amount of adaptation in predictive saccades.

Animal	Rightward			Leftward			Delay			Advance		
	Ang	Pos	Int	Ang	Pos	Int	Ang	Pos	Int	Ang	Pos	Int
E	0.38	0.28	*0.01	0.33	*0.01	0.21	0.08	0.13	0.40	0.32	*0.03	0.40
V	0.49	*0.00	0.11	0.29	0.09	0.75	0.07	0.35	0.60	0.08	0.90	0.60
W	*0.00	*0.00	0.48	*0.00	0.92	1.00	0.59	0.92	0.80	0.90	*0.01	0.80
All	*0.00	*0.01	0.51	0.07	0.21	0.86	0.07	0.54	0.91	0.71	0.58	0.91

Numbers indicate critical *p*-values derived from two-way ANOVAs. Asterisks denote statistically significant difference (*p* < 0.05). Ang, Angle; Pos, Position; Int, Interaction.

The middle column of Fig. 3 summarizes the data from three animals in temporal learning experiments. Similar to the representative experiment shown in Fig. 2b, latency of predictive saccades changed by 96 ± 20 ms (mean ± SD, *n* = 9, paired *t*-test, *t*_8_ = 14.2, *p* < 10^−3^) and −115 ± 44 ms (*t*_8_ = −7.8, *p* < 10^−3^) for the delay and advancement of target reappearance, respectively (Fig. 3, second row), while reactive saccade latency remained unchanged (mean differences: 0.8 and 7.0 ms, *t*_8_ = 0.25 and 1.9, *p* = 0.81 and 0.10, respectively). The temporal adaptation protocol did not alter the endpoints of either predictive or reactive saccades (Fig. 3, middle column, top and the third rows) during experiments with both delayed (mean differences: −0.18° and −0.13°, paired *t*-test, *t*_8_ = −0.96 and −1.1, *p* = 0.36 and 0.30, for predictive and reactive saccades, respectively) and advanced target reappearance (0.24° and 0.08°, *t*_8_ = 1.3 and 0.80, *p* = 0.23 and 0.45). Thus, our results showed that the animals could adjust the endpoint or timing of predictive saccades through learning and that adaptation of predictive saccades did not transfer to visually guided reactive saccades.

### Adaptation transfer from reactive to predictive saccades

**Figure 4:**
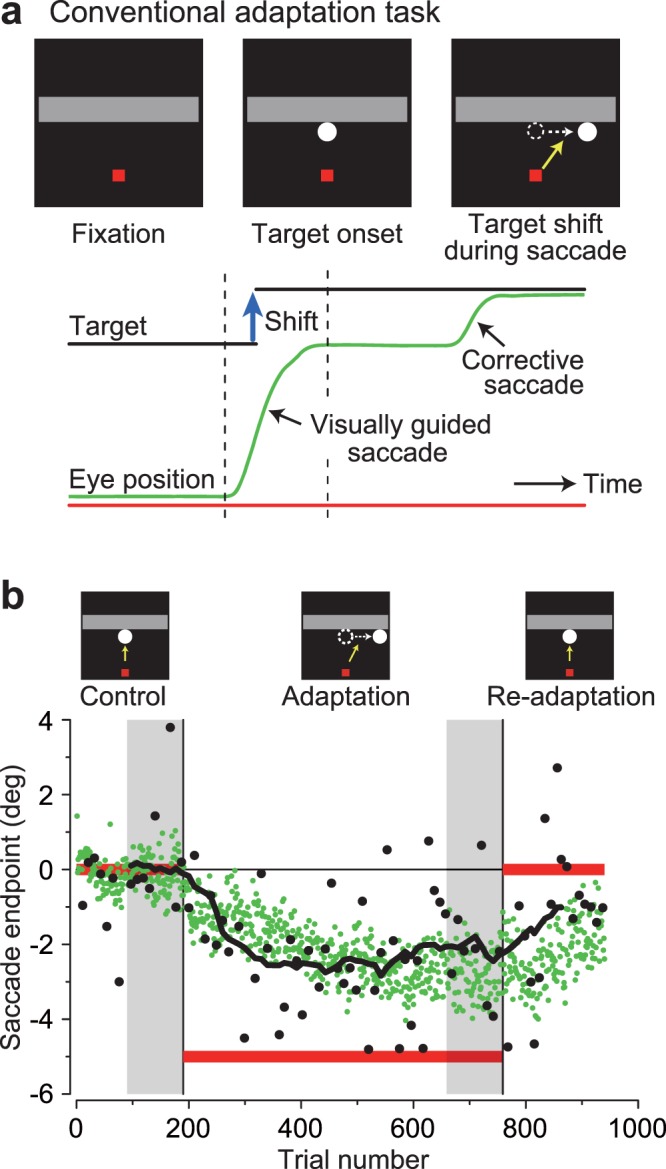
Effects of visually guided saccade adaptation on predictive saccades. (**a**) Saccade adaptation paradigm. Monkeys made an immediate saccade to a stationary target that suddenly appeared on the bottom side of the occluder. During the saccade, the target was relocated 5° horizontally. (**b**) Saccade endpoints relative to the initial target location are plotted as a function of trial number. Predictive saccade trials (black dots) were occasionally presented during the adaptation of visually guided saccades (green). Horizontal red lines indicate the final target locations. Quantification was based on reactive saccades indicated by gray shadow and the last 10 predictive saccades in the same blocks.

We have shown that the metrics of visually triggered reactive saccades were not altered during spatial adaptation of predictive saccades. We next examined whether the parameters of predictive saccades based on motion inference could be altered during the adaptation of reactive saccades. To induce adaptation of reactive saccades, the target suddenly appeared on the bottom side of the occluder and was relocated 5° horizontally during the targeting saccade (Fig. 4a). Figure 4b shows the time course of adaptation in a representative experiment in monkey V. As the target was relocated leftward in the adaptation block (horizontal red bar), saccade endpoints were gradually shifted in the same direction (green dots). In this experiment, saccade endpoints during the last 100 trials in the first and second blocks differed by −2.68° (unpaired *t*-test, *t*_181_ = 32.7, *p* < 10^−3^). For the occasional predictive trials, the saccade endpoints (black dots) and their running averages (15 consecutive trials, black trace) were gradually shifted leftward. On average, the endpoints of the last 10 predictive saccades during the first and second blocks differed by −2.36° (unpaired *t*-test, *t*_18_ = 3.0, *p* < 10^−2^). The adaptation transfer value from reactive to predictive saccades was 0.88 in the experiment shown in Fig. 4b. A similar result was obtained when the adaptation transfer was assessed by regression analysis (regression slope: 0.68).

We performed three experiments for each direction of adaptation (six sessions in total) for each monkey. The right column of Fig. 3 summarizes the data from three animals. Changes in the endpoints of visually guided saccades averaged 2.52° ± 0.75° (SD, *n* = 9, paired *t*-test, *t*_8_ = 10.1, *p* < 10^−3^) and −2.82° ± 0.33° (*t*_8_ = −25.8, *p* < 10^−3^) for the rightward and leftward adaptation experiments, respectively. During the adaptation of visually triggered saccades, the endpoints of predictive saccades were also altered in the same direction. On average, saccade endpoints moved 0.77° ± 0.66° (SD, *n* = 9, paired *t-*test, *t*_8_ = 3.5, *p* < 10^−2^) and –1.73° ± 0.93° (*t*_8_ = −5.6, *p* < 10^−3^) for rightward and leftward adaptation, respectively. Adaptation transfer from reactive to predictive saccades averaged 0.31 ± 0.26 (SD, *n* = 9) and 0.61 ± 0.33 for rightward and leftward adaptation, respectively. When the amount of adaptation transfer was quantified by computing regression slopes, the values averaged 0.18 ± 0.22 and 0.51 ± 0.36 for rightward and leftward adaptation, respectively. Thus, the adaptation of visually triggered saccades altered predictive saccades based on motion inference, whereas spatial adaptation of predictive saccades did not transfer to reactive saccades.

## Discussion

In this study, monkeys were trained to generate a predictive saccade for the location of target reappearance from behind a stationary occluder. When the target reappearance was systematically shifted in time, the latency of predictive saccades gradually changed while the saccade endpoints remained unchanged. When the target reappearance was shifted in space, the endpoints of predictive saccades gradually altered but the latency did not change. Furthermore, we found that adaptation of predictive saccades did not alter visually triggered reactive saccades, whereas adaptation of reactive saccades partially transferred to predictive saccades. These results indicate that the adaptation mechanisms for trajectory prediction and eye movements are hierarchically organized, which can be further examined in future studies at the level of neuronal activity in experimental animals.

**Figure 5:**
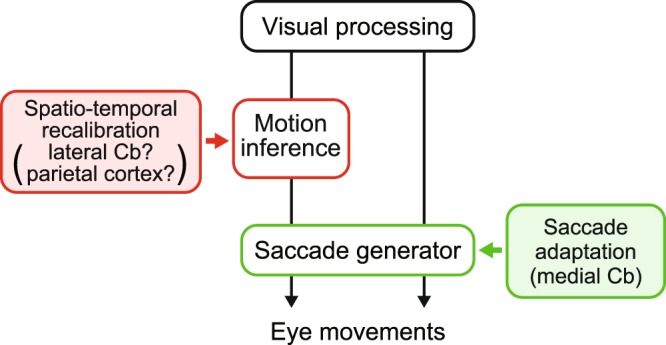
Diagram of possible neural mechanism. Visual information directly triggers reactive saccades or is further processed through the visual motion pathways to compute the location and timing of target reappearance for predictive saccades. Spatiotemporal recalibration of trajectory prediction takes place upstream of saccade gain adaptation which implicates the medial cerebellum.

Figure 5 illustrates a hypothetical block diagram for the relative locations of different types of adaptation. During the predictive saccade task, the visual motion signals are integrated to compute the expected location and timing of target reappearance; these prediction signals are then converted into the command of saccades. Systematic changes in the location or timing of target reappearance may render the predictive saccades seemingly inaccurate, which likely induces recalibration of the inference of target trajectory during occlusion. Because adaptation of predictive saccades did not transfer to visually guided saccades, the sites of spatial and temporal recalibration may reside upstream of saccade generating pathways (Fig. 5, left pathway). Conversely, during the visually guided saccade task, the target signal directly elicits a reactive saccade (Fig. 5, right pathway). When the target location is systematically shifted during visually guided saccades, the relationship between the retinal location of target images and the command of saccades gradually changes^26^. Because this saccade adaptation is known to take place far downstream in the system^27–30^, the adaptation of reactive saccades may transfer to the predictive saccades.

Our results indicate that the visual error for saccades does not always induce adaptation in the final common pathway for saccade generation. Many previous studies explored the relative location of the sites of saccade planning and adaptation in different behavioral contexts^31–35^. In particular, several laboratories have demonstrated that adaptation of visually guided saccades transfers to different types of saccades, including memory-guided saccades, delayed saccades, express saccades, scanning saccades and anti-saccades, while adaptation of memory-guided saccades transfers to delayed saccades only, which indicates that the site(s) of adaptation for memory-guided saccades is located upstream of that for visually guided saccades^30,31,36–40^. Neuronal mechanism underlying adaptation of visually guided saccades has been extensively examined in nonhuman primates; evidence shows that the vermal lobules V–VII in the medial cerebellum play a crucial role^41–46^.

Although both memory-guided saccades in literature and predictive saccades in the present study depend solely on the internal representation of invisible targets, they are different in several ways. The former requires spatial working memory of previously presented stationary visual stimulus, whereas the latter requires the inference of location and timing of target reappearance. Because the adaptation of memory-guided saccades neither transfer to targeting saccades^38^ nor alters visual, delay and saccade-related neuronal activity in the parietal cortex^47^, it might modify the conversion of visuospatial working memory into commands of saccades, which likely involve the frontal cortical networks. Conversely, the adaptation of predictive saccades in our behavioral paradigm may occur in the parieto-cerebellar networks that are relevant to the inference of target motion^7,8,10^. Recalibration of temporal prediction based on motion inference relies on the cerebellum^24^ and the effects of stimulus history on temporal discrimination depend on the signals in the parietal cortex^48^. Given that the parietal cortex integrates spatial information from multiple sources^49^ and that both the parietal cortex and the cerebellum are essential for timing^50–53^, the adaptation of predictive saccades based on motion inference might take place in the parieto-cerebellar networks.

When directing eyes immediately to a moving object, the brain must adjust motor outputs by computing the future target location at the time of saccade termination. Previous studies suggest a role for the brainstem, the cerebellum and the frontal cortex in this behavior^54–58^. Unlike such an immediate adjustment of interceptive saccades, our behavioral paradigm required temporal storage of target information as well as the adjustment of movement timing, which may recruit additional neuronal processes.

Because the location of target reappearance relative to the fixation point varied from trial to trial (among seven possible locations), the alteration of predictive saccades in the present study likely reflected changes in sensory prediction based on motion inference rather than an adaptation of specific motor commands. Previous studies examined neuronal activity when animals kept track of an imaginary moving target^1,10–12,59,60^ or covertly moved attentional focus with visible objects^61,62^. In our predictive saccade paradigm, monkeys might also imagine a moving target behind the occluder and continuously update their attentional focus during target occlusion^63^. Alternatively, because the target moved relatively fast (20°/s) so that the duration of target occlusion was short (580–710 ms), the animals may have predicted the location of target reappearance immediately after occlusion and then adjusted the timing of self-initiated saccades. In this case, the direction and speed of target motion might differently regulate the spatial and temporal parameters of predictive saccades, which might be subject to different adaptation mechanisms. This hypothesis of the dual processing could be examined in future studies using adaptation trials with combined spatial and temporal perturbations that induce coherent or incoherent perception of the changes in trajectory angles.

In summary, the present study showed that the predictive saccade for target reappearance was subject to spatial and temporal adaptation. These changes did not alter visually guided reactive saccades, whereas adaptation of reactive saccades partially transferred to the predictive saccades. These results suggest that there might exist recalibration mechanisms for the spatial and temporal aspects of predictive saccades, which are likely located upstream of the sites of saccade adaptation in the medial cerebellum.

## Methods

### Animal preparation

Three female monkeys (*Macaca fuscata*, 5–6 kg, monkeys E, W, V) were used. All animal protocols were evaluated and approved by the Animal Care and Use Committee of Hokkaido University and were in accordance with the Guidelines for Proper Conduct of Animal Experiments (Science Council of Japan, 2006). The animals were prepared for chronic experiments. The procedures for animal preparation are described in detail elsewhere^64^. Briefly, a head holding device was implanted on the skull using titanium screws and dental acrylic, and a search coil made of stainless-steel wire was implanted under the conjunctiva in the left eye. During each training and experimental session, the monkeys’ heads were securely fixed to primate chairs and their eye positions were recorded using the scleral search coil technique. The animals’ water intake was under daily control to motivate them to perform the behavioral tasks.

### Visual stimuli and behavioral paradigms

Experiments were controlled by a Windows-based real-time stimulus presentation and data acquisition system (Reflective Computing). Visual stimuli were presented on a 24-inch cathode-ray tube monitor (60 Hz) positioned 38 cm away from the eyes (subtended 69 × 52° in visual angle). All experiments were performed in a darkened booth.

The animals were trained in two behavioral tasks with different adaptation protocols. In all experiments, a stationary gray rectangle (10 × 52°, ‘occluder’) was displayed at the center of the screen throughout trials. Each trial began with the appearance of a fixation point (FP, red 0.5° square) in the lower half of the screen and ended with a drop of juice reward for correct performance or beep sounds for errors. The FP was always presented along the vertical meridian and 12° below the bottom side of the occluder. In the predictive saccade task (Fig. 1a), a target spot (white circle, 0.5° diameter) appeared above the occluder and moved along a straight path at 20°/s. For each trial, the angle of the target path was randomly selected among 45, 60, 120 and 135° from the horizontal meridian. After a 500-ms excursion, the target was occluded behind the rectangle. The duration of target occlusion was approximately 710 and 580 ms for the 45° (135°) and 60° (120°) trajectories, respectively. The horizontal location of target reappearance was randomly selected among ±15°, ±10°, ±5°, or 0° from the vertical meridian and the target trajectory before the occlusion was adjusted accordingly in each trial. Consequently, there were 28 combinations of initial target locations and trajectory angles (seven exit locations with four angles of the target path). The initial target location varied ±32.1° horizontally relative to the screen center and was 7.1° or 8.7° above the top of the occluder. Monkeys were trained to make a predictive saccade to the position where the target would reappear on the bottom side of the occluder. To prevent monkeys from generating immediate saccades following target occlusion, trials were aborted for early fixation break (>3°) that occurred >200 ms before target reappearance. To obtain a liquid reward, the animals were required to *terminate* saccades to the expected location (<3° accuracy) within ±150 ms of target reappearance. Because saccade duration was approximately 50–70 ms, the animals needed to *initiate* saccades earlier than 80 ms following target reappearance. During training sessions, the target continued to move after its reappearance; however, it remained stationary during experimental sessions to exclude possible confounding effects of retinal image motion around the time of predictive saccades. Once the animals were successful in >70% of the trials, they participated in the adaptation experiments (see below).

To induce spatial and temporal learning of trajectory prediction, we devised two adaptation paradigms. In the spatial adaptation paradigm (Fig. 1b), the location of target reappearance was displaced 5° horizontally, without changing the initial target trajectories above the occluder. The direction of displacement was either leftward or rightward and was consistent in each experimental session. In the temporal adaptation paradigm (Fig. 1c), the target reappearance was delayed or advanced by 200 ms. During these adaptation trials, both the spatial and temporal requirements for correct performance were relaxed to prevent the animals from altering behavioral strategy based on reward outcome. In the spatial adaptation trials, the criteria for correct saccades were widened so that the animals were rewarded for learned saccades to the 5° target reappearance displacement (8° horizontal and 3° vertical). In the temporal adaptation trials, the criterion for correct saccade timing was relaxed from ±150 ms to ±350 ms. In each experimental session, trials were presented in a sequence of control, adaptation and re-adaptation (control) blocks, which typically contained 200, 600 and 200 trials, respectively. Each adaptation condition was repeated three times in each monkey and different adaptation experiments were presented randomly on separate days. We collected the data with different adaptation protocols (18 sessions) in a month for monkey E, but it took several months for the other monkeys.

To examine adaptation transfer from predictive to reactive saccades with similar direction and amplitude, visually guided (reactive) saccade trials were occasionally presented throughout the blocks (1 out of 17 trials). In these probe trials, a stationary target spot suddenly appeared and the animals were rewarded for making an immediate targeting saccade (with 3° accuracy) within 300 ms. The target location was always along the bottom side of the occluder and was randomly selected among ±10° and 0° from the vertical meridian. To examine adaptation transfer from reactive to predictive saccades, we also presented the conventional saccade adaptation paradigm^26,65^ in separate sessions. In the adaptation trials, the target presented on the bottom side of the occluder (±10° or 0° from the vertical meridian) was shifted 5° horizontally during the targeting saccade (Fig. 4a). In this case, the predictive saccade trials with occluded motion were occasionally presented as probe trials during the adaptation experiments (one out of eight visually guided saccade trials).

### Data analysis

Data were digitized and sampled at 1 kHz and stored in files during experiments. Further offline analysis was performed using MATLAB (MathWorks). Eye position signals were taken directly from the eye coil electronics and horizontal and vertical eye velocity were obtained through digital differentiation. Saccade initiation was defined as the time when angular eye velocity exceeded 40°/s and saccade termination was when it went returned below 20°/s.

The level of adaptation was quantified by computing the changes in horizontal saccade endpoint and latency measured during the last 100 trials in the first (control) and the second (adaptation) blocks (shaded areas in Figs. 2 and 4b, respectively). For saccade latency, we measured the time between the expected timing of target reappearance and saccade *initiation*, although the animals were reinforced to adjust the timing of saccade *termination* during the predictive saccade trials (see above). This resulted in an approximately 90-ms advancement of saccade timing relative to target reappearance (Fig. 2, lower panels). Because saccades generated later than 80 ms following target reappearance might be visually guided^66^, those trials were excluded from further quantitative analyses. The percentage of excluded trials averaged 0.44 ± 0.51% (*n* = 45 sessions, ranged from 0.0–2.0%), except for the temporal advancement paradigm. During the temporal advancement adaptation, those numbers increased because the target reappeared 200 ms earlier than the expected timing (12.0 ± 8.3%, *n* = 9, ranged from 0.3–28.8%). For this condition, we obtained similar results even when the late saccades (>80 ms) were included for the analysis.

For each experiment, the level of adaptation was assessed by computing the learning gain, which was defined as the change in saccade parameters (either horizontal endpoint or latency) divided by the changes in the location or timing of target reappearance (either 5° or 200 ms). Unpaired and paired *t*-tests were applied to evaluate the amount of adaptation in each experiment and across multiple experiments, respectively. We also quantified adaptation transfer between predictive and reactive saccades in each experiment. The changes in reactive saccades during adaptation of predictive saccades were assessed for the last 10 visually guided saccade trials in each block. Similarly, the changes in predictive saccades during the adaptation of visually guided saccades were measured from predictive saccades in the last 10 motion inference trials in the block. Additionally, we also performed a regression analysis between predictive and reactive saccades during the course of adaptation. To do this, the change in the horizontal endpoint of each reactive (predictive) saccade was paired with that of the preceding predictive (reactive) saccade and the regression slope was taken as the amount of adaptation transfer. To minimize the effect of outliers for the regression analysis, we used the S estimator^67^. Generally, regression analysis methods with robust estimators iteratively determine a weight for each data point so that the outlier has a smaller weight. The S estimator has a higher resistance to outliers by introducing the scale parameter on the dispersion of estimation error. A two-way analysis of variance (ANOVA) was used to detect the possible confounding effects of the location and the angle of the target trajectory on the magnitude of learning (Table 1). Details of statistical tests are reported in the relevant text in the Results.

## Data Availability

Data supporting the findings in this study are available from the authors upon reasonable request.
